# Development of telemedicine in the Czech Republic from patients’ and other key stakeholders’ perspective

**DOI:** 10.3389/fpubh.2023.1202182

**Published:** 2023-10-23

**Authors:** Jolana Kopsa Těšinová, Karolína Dobiášová, Zdeněk Dušek, Alena Tobiášová

**Affiliations:** ^1^Institute of Public Health and Medical Law, First Faculty of Medicine, Charles University, Prague, Czechia; ^2^Ernst & Young, s.r.o, Prague, Czechia

**Keywords:** development of telemedicine, patient organizations, community, patient and public involvement, patient interest, stakeholders, telehealth

## Abstract

Telemedicine is a way to improve healthcare outcomes with greater efficiency for both patients and care providers. The great potential of digital technologies also lies in strengthening the patient-centered approach. The early successes and benefits of telemedicine in the Czech Republic, amplified by the COVID-19, have contributed to the fact that wider implementation of telemedicine is already generally supported at the expert and public levels. Our research focuses on the identification of key issues in the implementation of telemedicine and the challenges of telemedicine in the future, from the perspective of patients and other stakeholders. The study is based on a qualitative research approach, combining focus groups with key stakeholders, patient panels and expert panels (2021–2022). The lack of rules and uncoordinated development of various activities proved to be the main barriers to the integration of telemedicine in the health system. This regulatory uncertainty can generate a number of problems in the patient–doctor relationship in practice, including ethical ones, and can also lead to inequalities in access to healthcare and affect the overall quality of care provided. Furthermore, it has been shown that patients’ interests in the implementation of telemedicine are: 1. a predictable and reliable framework that guarantees them certainty and security in the provision of telemedicine services, 2. telemedicine solutions that increase the availability and efficiency of the care provided while bringing comfort, and 3. user-friendly and simple solutions. At the same time, patients want to understand the new environment and be active participants in the process of digital innovation, including the practical implementation of telemedicine. The research team has developed recommendations for further developments in the implementation of telemedicine that reflect the patient’s interest and can be implemented at three levels – the health system, institutional, and community level. In countries with a well-developed and institutionalized patient movement, the community level can be represented by patient organizations, thus becoming the link between telemedicine policy making and implementation at the individual level of healthcare provision. For the further development of telemedicine, the development of a national strategy involving all key stakeholders, including patients, in the implementation has proven essential.

## 1. Introduction

In the last two decades, health policy makers, influenced by a number of factors such as the demographic ageing of the population and the continuous increase in healthcare spending, have been trying to introduce new approaches in healthcare with a focus on digital solutions (1). Also contributing to this is the pervasive development and spread of information communication technology (ICT) and digital technologies in healthcare management and delivery (2). The COVID-19 epidemic also contributed to an unprecedented acceleration in the adoption and spread of ICT and digital solutions within health systems (3, 4).

Digital transformation was an important issue even before the coronavirus pandemic but, during the crisis, the development and implementation of various modern technologies accelerated, making the digitization of healthcare an overwhelming priority for most countries. In particular, there has been an increase in the use of telemedicine, which has become indispensable in ensuring continuity and accessibility of healthcare during epidemics (3).

In the Czech Republic, the legislative development of eHealth is only at the beginning, and despite the adoption of the Electronization of Healthcare Act (5), it is still among the countries with a lower level of digitization of healthcare processes, with the exception of partial aspects of digitization (e.g., e-prescription).

One of the decisive factors for the adoption of telemedicine by patients and healthcare providers is the reimbursement policy for telemedicine solutions (6–8). Considering that the healthcare system in the Czech Republic is based on compulsory health insurance, which guarantees equal access to healthcare and covers a wide range of services (9, 10), finding an optimal and sustainable way of its reimbursement from public health insurance is essential for the future use and development of telemedicine.

The main goal of health systems is to promote, restore, and maintain the health of the population, and therefore they should respond to the needs and expectations of the public. This is the reason why in the last decade there has been a growing effort to involve patients and the public (PPI) in health policy decision making processes (11). Different countries use a variety of tools, policies and interventions to systematically improve the position of the patient in the health system (11, 12).

In the Czech Republic, too, there has been a greater involvement of patients in health policy decision making in recent years. Since the 1990s, the first patient organizations were established, laying the foundation for the patient movement. There are currently around 140 patient organizations in the Czech Republic (13). The turning point for the development of PPI in the Czech Republic was the adoption of the Health Services Act in 2011 (14) which, for the first time, defined patients’ rights at the level of national legislation (9).

**Figure 1:**
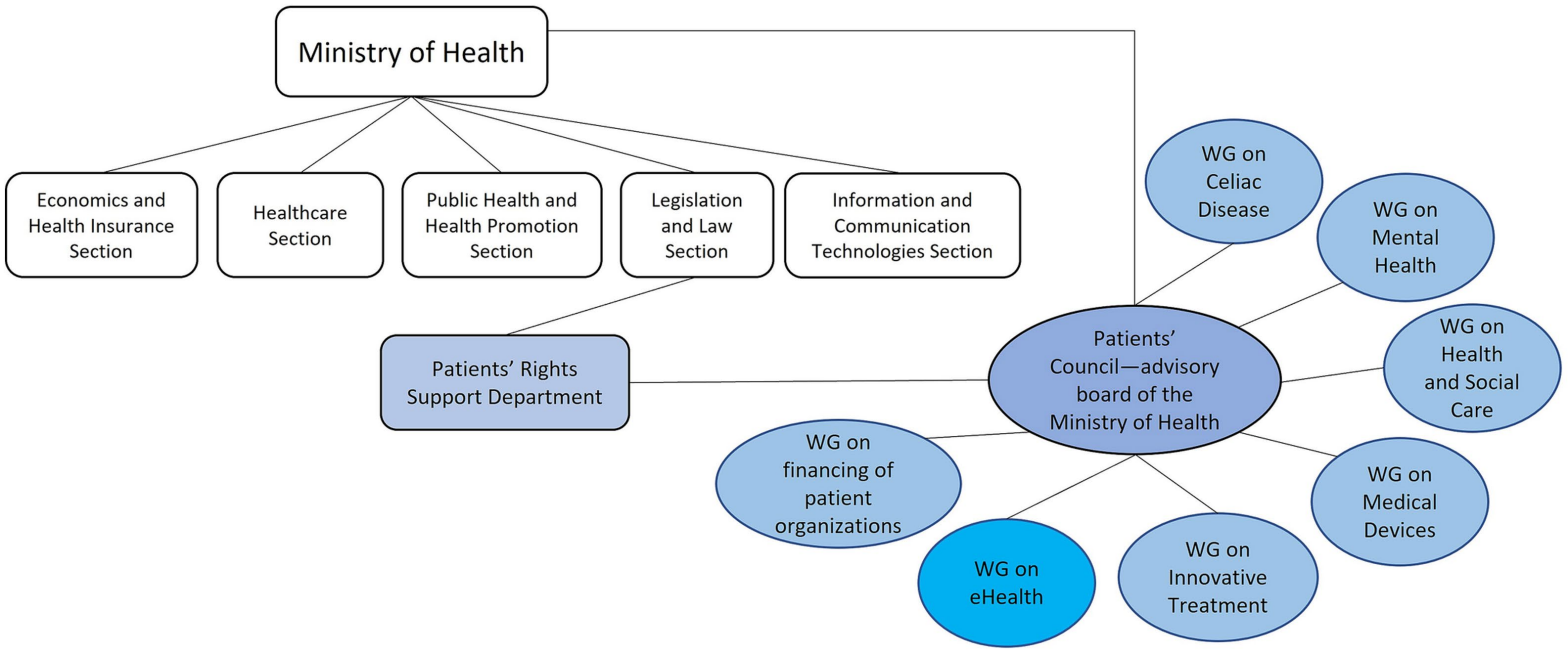
Patients’ council and working groups.

The establishment of the PPI as a permanent part of the organizational structure of the Ministry of Health (Figure 1) in 2017 was crucial for the institutionalization of PPI (15). In the same year, the Patients’ Council was established (16), which is a permanent advisory body to the MoH, with as its main mission to promote patients’ rights, including participation in the legislative process. The Patients’ Council currently has seven permanent working groups, one of which is explicitly dedicated to eHealth (Figure 1) (12).

Another important milestone of PPI was in 2021, when the patient organization was defined at the level of law and thus the involvement of patients in decision making processes was significantly strengthened (17). In the same year, the National Association of Patient Organizations (NAPO) was established, bringing together patient organizations focused on all types of diseases and disabilities in the Czech Republic. It carries out advocacy and awareness-raising activities and represents patients vis-à-vis state authorities. One of NAPO’s main priorities is the digitalization of healthcare (13).

The aim of our research is to identify key issues in the implementation of telemedicine in the Czech Republic, and the challenges of telemedicine in the future from the perspective of patients and other stakeholders.

## 2. Methods

**Figure 2:**
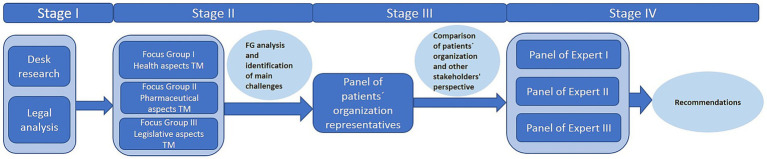
Exploratory qualitative research design.

The study is based on an exploratory qualitative research approach with regard to the unexplored area of the implementation of telemedicine in the Czech Republic. The research was divided into four consecutive stages (see Figure 2).

### 2.1. Stage I

In stage I, a desk research was implemented, where team members worked with the literature, available statistical data, and health policy documents. In parallel, an analysis of legislation related to telemedicine in the Czech Republic in the context of European Union legislation was carried out. Based on the findings from this first stage of the research, a matrix of open questions was prepared for the follow-up stage 2 of the research.

### 2.2. Stage II

In stage II of the research, three focus group (FG) discussions (18) were conducted with key stakeholders of telemedicine implementation in the Czech Republic. Each FG had a different thematic focus (see Table 1). Informants were selected by purposive sampling to cover different areas of telemedicine implementation (19): physicians, patients, pharmacists, health care managers (of health insurance companies and health care facilities), officials of relevant government institutions (e.g., Ministry of Health). The institutional representation of informants is shown in Table 1. A total of 32 stakeholders were involved. The aim of the FG discussions was to identify problems related to the implementation of telemedicine in practice, to place them in the broader context of the Czech health system, to structure them, and to identify challenges for telemedicine in the Czech Republic in the future. All FG discussions were performed virtually in August 2021 using online meeting platforms Zoom and lasted approximately 2 h each. Online FG discussions allowed for a wider geographical coverage and a greater diversity of informants (20). At the beginning of each FG, all participants were briefed on the focus and objective of the research. With the consent of all participants, FG discussions were recorded. FG recordings were transcribed verbatim, anonymized, and subjected to thematic analysis (21). Based on the analysis of the FG discussions, the research team identified and described ten core areas of telemedicine implementation. The FG results formed the basis of the next stage of the research.

**Table 1:** Institutional representation of stakeholders in focus group discussions.

Focus group I health aspects		Focus group II pharmaceutical aspects		Focus group III legislative aspects	
Informant	Institution	Informant	Institution	Informant	Institution
FG I-I1	University hospital	FG II-I1	State administration	FG III-I1	State administration
FG I-I2	Medical Institute	FG II-I2	Medical Institute	FG III-I2	Health insurance fond
FG I-I3	University hospital	FG II-I3	Pharmacist Society	FG III-I3	Health insurance fond
FG I-I4	Medical Society	FG II-I4	Pharmacy operator	FG III-I4	State administration
FG I-I5	Clinical center	FG II-I5	Pharmacist Society	FG III-I5	University hospital
FG I-I6	Medical Society	FG II-I6	Pharmacist Society	FG III-I6	University hospital
FG I-I7	Medical Society	FG II-I7	Patient organization	FG III-I7	Patient organization
FG I-I8	Patient organization	FG II-I8	Patient organization	FG III-I8	Patient organization
FG I-I9	Patient organization	FG II-I9	Pharmacist Society	FG III-I9	Medical Society
FG I-I10	Medical Society	FG II-I10	Pharmacist Society	FG III-I10	Pharmacist Society
FG I-I11	Medical Society			FG III-I11	Medical Society

**Table 2:** Background information on participants in the expert panel.

Institution	Number of experts
Society of Physicians	3
Health insurance companies	3
Drug and medical technology manufacturers	3
Consulting and advisory firms	3
Patient organizations	2
Pharmacists’ societies	2
Hospitals	2
State administration	2
Treatment institutes	2
Academia	2

### 2.3. Stage III

In stage III (September/October 2021) of the research, a panel of patient organization representatives was assembled to assess and supplement the results of FG discussions with stakeholders on the perspective of the patient. In recent decades, there has been a growing debate about patient and public involvement (PPI) in health care decision-making (11) and health research (22, 23). Various methods are used to involve patients, such as patient panels, patient and public involvement panels, patient advisory boards, citizen juries, advisory committees, etc. (23). In addition, representatives of patient organizations, who usually have long experience in chronic disease management, can provide valuable information on what works and what does not work in practice. In addition, existing evidence shows that involving patients in the analysis, design and implementation of health policy increases patient confidence in and acceptance of new decisions. For these reasons the National Association of Patient Organizations (NAPO) was approached to help select members for the patient panel. Selected NAPO members were invited to become members of the patient panel. Here, patients did not figure as research participants but as partners of the research team (23). All panel members were made aware of the research objectives and agreed to participate as part of the patient panel. They were not remunerated for their participation in the panel. The patient panel included 6 representatives from various NAPO-affiliated patient organizations. All patient panel members had experience in the area of telemedicine implementation. One of the panelists was also a member of the Patient Council of the Ministry of Health, and two panelists were also members of the Patient Council e-health working group (see Figure 1). The patient panel received a report with the results of the focus groups with stakeholders in October 2021 for review. The Patient Panel discussed the results initially in the presence of the research team leader, who facilitated the discussion. The discussion lasted approximately 3 h. Subsequently, the patient panel met once more without an external facilitator, and members of the panel worked together to develop a patient perspective on the results of the focus groups. This position paper included both patients’ concerns about some aspects of telemedicine implementation and patients’ expectations for telemedicine in the future. Representatives of NAPO presented their position at a professional conference on telemedicine (24).

Members of the research team then compared the results of the FG discussions with the views of the patient panel, identifying areas where the patient’s perspective differed from that of other stakeholders, and areas where it was consistent. This comparison formed the basis for the follow-up stage IV research.

**Table 3:** Identification of the “patient interest.”

Key areas of telemedicine implementation	Focus group	Patient panel	Patient interest
	Identification of the problem/challenges of TM	Identification of the problem/challenges of TM	
Legislative environment	Lack of legislative definition of TM	Uncertainty if TM is legal	Responsibility of health service provider to be clearly defined
	Defining TM in law	Support for legislative definition	
Guidelines	Absence of guidelines	Concerns about whether the approach is professionally correct	(Published) guidelines available for patients (e.g., database)
	Development of guidelines by specialization	Support the development of best practices	
Technology and applications	Absence of rules for technological solutions	Concerns about invasion of privacy and safe provision of care	A database of safe technology solutions (health apps) available
	Define a standard and address the safe use of health applications	Promote a standard of secure technology solutions	
Communication and data sharing	Different perspectives on remote communication, with particular emphasis on formal and security aspects	Worries of suppressed autonomy of decision making, uncertainty of communication in a new and unfamiliar environment	A digital communication standard as a basis for communication between doctors and patients in an online environment
	Promote discussion among experts on remote communication	Support for the creation of rules for communication	
Organization of care and conditions of provision	The absence of rules for the inclusion of TM in the organization of healthcare and the conditions for the provision of remote care	Concerns about inconsistent and confusing settings between providers, concerns about the availability and ability to use technology for communication	Provider’s awareness of TM interventions, enabling online bookings, making available guides for individual TM solutions and ensuring education from specific providers
	Define a time pool for remote care, support optional TM settings	Support for the organizational set-up and unification of TM conditions on the provider side, taking into account the specifics on the patient side	
Electronic pharmacy	Insufficient use of e-pharmacy tools and collaboration between doctors and pharmacists	Concerns about the limited availability of medicines for certain patient groups and their safe dispensing	Retaining autonomy in deciding how medicines are dispensed, enabling the whole remote end-to-end cycle online, and expanding it to include distance dispensing
	Support the development of e-pharmacy tools, redefine the relationship between doctors and pharmacists, lead the discussion on remote dispensing	Support for the development of e-pharmacy tools, support for remote consultation by pharmacists and remote dispensing of medicines	
Reimbursement of telemedicine solutions	Absence of conditions for TM entry into reimbursement	Concerns that providers will not be motivated to provide TM	Transparent process with the participation of representatives of patient organizations
	Define conditions for inclusion of TM in reimbursement, including with regard to their effectiveness	Support for TM to enter into reimbursement	
Education of healthcare professionals	Lack of training programs, low digital literacy of health professionals	Concerns about the safe use of digital technologies by healthcare professionals and the proper provision of remote care	To involve patient organizations in the education of health professionals
	Support training of health professionals and defining their new competences	Support the training of healthcare professionals and defining their new competences	
Patient education and awareness	Low health and digital literacy of patients	Concerns about poor access and quality of care due to lack of understanding of TM	Involvement of patient organizations in patient and public education in the field of TM
	Support for activities and programs to increase their literacy	Support for activities and programs to improve these	
Prevention and health promotion	Under-utilized potential of ICT in prevention and health promotion	Untapped benefits in terms of health and increased quality of life (comfort) for patients	To address in a systemic way in society
	Support for ICT tools that increase patient compliance	Support for use of ICT tools that increase patient	

### 2.4. Stage IV

The aim of stage IV of the research was to create recommendations for further development of telemedicine implementation in the Czech Republic, taking into account “the interest of patients.” The members of the research team identified and invited experts from various fields who are extensively involved in the implementation of telemedicine in the Czech Republic. The Multidisciplinary Panel of Expert included 24 experts from different fields so as to represent a wide range of relevant opinions and expertise. Their institutional background is shown in Table 2. They included representatives of physicians and pharmacists, patient organizations, health care managers, representatives of insurance companies, lawyers specializing in health care, government officials and health policy makers, researchers, producers and distributors of drugs and health technologies. A total of three half-day (approximately 3 h) expert panel meetings were conducted in February, May and October 2022 in a face-to-face format. All experts were briefed in advance on the results of the research to-date (stage II and III), i.e., the findings of the focus group discussions and patient opinions. The experts were also presented with an overview of the implementation of telemedicine in selected countries and the status of domestic and EU legislation on telemedicine and e-health in the form of powerpoint presentations. This information was used as a stimulus for discussion. At each session, a number of open questions on telemedicine were presented to the panel of experts so that the patients’ point of view was always reflected. The discussion was moderated by one professional moderator and two members of the research team. Two members of the research team took notes of the discussions. Based on the experts’ discussion the research team formulated recommendations for the further development of telemedicine with respect to preserving “patients’ interests” (Table 3).

## 3. Results

### 3.1. Legal analysis

The research also included an analysis of the legal aspects of distance (remote) medicine using ICT reflecting the findings from the empirical data. It is apparent that the provision of health services in the Czech Republic is only possible on the basis of an authorization to provide them, which must correspond to the type and form of health care provided according to the Health Services Act (14).

The current concept of providing health services presupposes the personal (physical) presence of the patient in a health care facility, or the physical presence of the doctor in the patient’s own social environment (e.g., in the context of home care). To some extent, consultations may be provided by remote access, but without being defined in more detail by law. This concept thus makes it considerably more difficult for healthcare to be provided by remote access via ICT, The provision of healthcare only through a ‘virtual’ provider who would not have a healthcare facility is completely excluded.

Despite the adoption of the Electronization of Healthcare Act (5), the field of remote care remains without direct legislative support. The same rules apply to telemedicine as to the provision of healthcare in general, i.e., it must be provided at the appropriate professional level (lege artis), i.e., according to the rules of science and recognized medical practices, respecting the individuality of the patient, taking into account the specific conditions and objective possibilities. The current legislation does not provide sufficient legal certainty for the provision of telemedicine services.

### 3.2. Focus groups with stakeholders

By analyzing the content of the transcripts of the focus group discussions, ten key areas for the implementation of telemedicine in the Czech Republic were identified: legislative environment; guidelines; technologies, applications and safe environment; communication and data sharing; organization of care and conditions of provision; electronic pharmacy; reimbursement of telemedicine solutions; education and competences of healthcare professionals; patient education and awareness; prevention and health promotion. Within these key areas, the research team focused on the main issues and challenges of telemedicine in the Czech Republic from the perspective of the interviewed stakeholders.

#### 3.2.1. Legislative environment

The FG participants agreed that the legislative environment is a key factor for the successful development of telemedicine (25). They also pointed to the problematic current concept of health service provision in the Czech Republic, which, with exceptions (e.g., second opinion consultation), assumes the personal presence of the patient in the health care facility, or the physical presence of the health care professional in the patient’s own social environment.

*“Rather, it is assumed that the Health Services Act has been traditionally conceived as the very law that regulates the health care that we primarily knew in 2011, when it was passed. It is care that is provided in a health facility, with exceptions as a visiting service or a preventive service in the field.” (FG III-I1).*

The field of telemedicine remains without specific legislation in the Czech Republic, despite the adoption of the Electronization of Healthcare Act.

*“The Law on the Digitization of Healthcare rather introduces new elements for communication in the digital space to make it safe both in the technical sense and in the sense of who communicates with whom.” (FG III-I1).*

Thus, the same rules apply to the provision of telemedicine services as to the provision of healthcare in general, i.e., they must be provided at the appropriate professional level (de lege artis). However, this regulation does not fully reflect the specificities of remote contact.

*“Today, we do not have the word telemedicine in Czech law, but this does not mean that it is not regulated. It is regulated by general regulations both for medicine and for the provision of healthcare services, and more broadly for the use of IT tools, medical devices, privacy and cybersecurity.” (FG III-I3).*

The stakeholders agreed on the necessity of defining a basic legislative framework for telemedicine and the use of ICT with gradual follow-up professional and other legal regulation.

*“Giving the basic legal framework and testing where it makes sense to develop those services in the future, and where some follow-up regulation will be needed.” (FG III-I1).*

*“There is definitely a need for some further regulation to enter into this, both by legislation and, of course, by having medical experts define what type of healthcare is still Lex Artis. This must be done by experts in the field.” (FG III-I3).*

At the same time, they expressed concerns about robust legislation that could hinder the development of telemedicine solutions.

*“This legislation must not hinder progress.” (FG III-I1).*

Therefore, minimalist legislation with broadly defined rules and a clear definition of responsibilities was preferred.

*“The regulations must be minimalist and progressive. Gradual steps are, in my opinion, far better in this respect.” (FG III I6).*

*“It is necessary to address the responsibility for the outcome of the diagnosis made; where the limit for determining diagnoses is, that is a question for the doctor.” (FG III-I5).*

In defining the rules of telemedicine, the need for cooperation between the Ministry of Health and professional societies was also emphasized (see Guidelines).

#### 3.2.2. Guidelines

FG participants agreed that areas, disciplines and procedures appropriate for remote care must be described through clinically guidelines (professional standards).

*“It is up to the experts to clearly declare what part of medicine is suitable to be implemented by this modern innovative tool, i.e., telemedicine.” (FG III-I2).*

In the Czech Republic, the current guidelines only sporadically address telemedicine (26). All stakeholders agreed that the legislative anchoring of telemedicine in law should contribute to its greater development.

*“There is no one area for telemedicine, and it depends on the field of medicine communicating with the patient. There will be different opportunities in oncology, different opportunities in GP. It is imperative that the option is there, but it will vary greatly by medical field and by specialization.” (FG I-I6).*

They stressed, however, that the appropriateness of using telemedicine tools, even when the conditions implied by the guidelines are met, must be assessed by the physician on an individual patient basis.

*“I would venture to say that it will probably always be at the discretion of the doctor. He/She must have the final say, whether this is something that can be dealt with remotely, or must be dealt with face to face.” (FG I-I4).*

The development of clinical guidelines should serve as a basis for the development of innovative telemedicine interventions that can sustain quality of care in times of pandemic (or other crisis situations).

*“Telemedicine is the future, but especially for chronic patients. In acute care, telemedicine is just a small addition that can be used in some crisis situations like a pandemic.” (FG I-I5).*

#### 3.2.3. Technology, application, and safe environment

The FG participants agreed that the creation of a digitally secure environment for communication, sharing and data compatibility, as well as their control, is a prerequisite for the wider implementation of digital technologies in health care models.

*“For the most part, the focus should be on cyber security, which is still largely overlooked.” (FG I-I3).*

They pointed out the absence of rules for technological solutions enabling communication with patients via remote access and its impact on practice.

*“It is the lack of standardization and the absence of a law. Today, if those do not exist, there is no way to ask the patient to connect with each doctor differently. Each person uses what is nearest to them. If you know how to use WhatsApp, and you ask the doctor and you have his number, it is easier for him to send you something, or for you to send something to the doctor.” (FG I-I3).*

Complicating matters in the Czech Republic is the possibility of legal clinical use of data from devices that are not approved medical devices. The issue of certification of data obtained from medical applications has not been resolved.

*“It is supplementary data that we cannot yet consider certified, but it’s just a matter of time. I’m sure legislation will include these more in the clinical process.” (FG I-I3).*

Stakeholders agreed that systems (technologies) supported should be simple, safe and also affordable for providers and patients.

*“We need to make sure that those systems are simple, secure, inexpensive, and also that the information systems operators open them up for inexpensive solutions.” (FG I-I6).*

They recommended defining uniform technical and security standards for the use of digital platforms by individual healthcare providers.

*“There should be some way of defining how the patient should connect with the doctor.” (FG I-I8).*

They also recommended defining rules for the use of non-certified health apps, including rules for sharing data collected from these devices.

*“Can I trust those values? There’s going to be a problem with standardization. Those are obviously things we are going to have to address…” (FG I-I4).*

Creating a uniform and transparent environment for the use of telehealth services and setting up certification systems for telemedicine solutions is perceived by experts as a task for the state.

*“We need to have a framework within which to operate. We perceive that the one who will set the framework will be the Ministry of Health.” (FG III-I2).*

#### 3.2.4. Communication and data sharing

Respect for patient autonomy is a key requirement of current ethical and legal codes (27). Fulfilling the principle of autonomy is only possible on the basis of proper patient education. FG participants agreed that communication in healthcare is a problem in the Czech Republic in general.

*“It happened to me repeatedly: a patient who had come from a specialist telling me that I was the first one in six months to a year to listen to them. It’s terribly important to put demands on the education of doctors in the area of communication with patients, not just the specialist component.” (FG I-I11).*

They also agreed that telemedicine limits, or even negates, some forms of communication that are essential for determination of proper treatment.

*“The physical presence of the patient in the office is extremely important. We can read the patient’s posture, their attitude, assess their psychological aspects much more easily when we have them next to us. Of course, a flat screen is a kind of substitute, but, again, there are many things we cannot see.” (FG I-I1).*

FG participants pointed out the possibility of using modern technology in educating the patient about possible treatment procedures.

*“If the patient does not have a certain level of health literacy and, at the same time, is under a lot of stress, the amount of information at one time can be a big problem for them, and they may feel some discomfort and prefer not to express their opinion at that moment. And this is where modern technology can help a lot: presenting the patient with treatment options online first, and then, already specifically educated, discussing the most appropriate treatment with the doctor.” (FG I-I2).*

Stakeholders pointed in particular to the risks associated with the security of personal data and the invasion of privacy in remote communication. They also expressed concerns about potential implications for legal liability in relation to poor communication, however without proposing solutions.

*“There is a pronounced risk of misuse or invasion of privacy and abuse of data protection.” (FG III-I1).*

*“The responsibilities of the doctor and the patient when communicating remotely must be clearly established.” (FG III-I5).*

#### 3.2.5. Organization of care and conditions of providing

Successful implementation of telemedicine requires not only changes in the technological infrastructure, but also in the organization of healthcare and the organization of healthcare professionals. FG participants agreed that the time pool of health care providers devoted to telecare during office hours should be clearly defined.

*“At the moment, we cannot define the time that a doctor who wants to provide this service should schedule in his/her office. It should be about what the logistics of those services they will provide, as well as scheduling some visiting services. The doctor should, probably within their office hours, schedule teleconferencing to address their clients’ problems.” (FG III-I3).*

Telemedicine should not lead to unrestricted use of health care that would disproportionately increase the workload of physicians.

*“In the context of telemedicine, anyone can write an email or a message to their doctor at any time. The amount of information that comes in this way is so vast. It’s similar with phone calls. Some regulation is needed and we need to talk about how to regulate that.” (FG I-I4).*

When incorporating telecare, the organizational capabilities and operational conditions of a particular health service provider should be respected.

*“The problem with telemedicine is some division of working hours. There should just be some time pool that needs to be dedicated to it. The idea that a doctor is constantly online and constantly communicating with a patient and that he/she is basically available on call at any time, which is what a lot of patients imagine telemedicine to be, is completely wrong.” (FG III-I5).*

Stakeholders agreed that the setting up of telecare by healthcare providers should be optional, not an obligation for all participants.

*“We see providing this service as an option that we would not want to make mandatory; medicine is primarily about patients seeing doctors in person, but this is an option, and it will as such depend on the experience of the doctor and their willingness to provide this service.” (FG III-I2).*

They also pointed out that new models of care using ICT may introduce new risks, exacerbate existing health inequalities and, for a certain segment of the population, reduce access to healthcare. Therefore, the specificities in terms of the patient’s health status (e.g., immobility), their particular capabilities (e.g., availability of technology), but also their social background (e.g., cooperation of family members) should be taken into account.

*“There are people who are socially vulnerable, people with disabilities, older adult, or maybe just less technologically adept, even some younger people. These people are there and we have to provide proper health care for them, and telemedicine is not going to be an appropriate way of providing health services for them.” (FG III-I1).*

The setting, quality, and sustainability of telemedicine services must be consistent with the goal of universal access to health care and should also promote continuity, coordination of care and a multidisciplinary approach.

#### 3.2.6. Electronic pharmacy

Digitization in the Czech Republic has long been in effect, mainly in the field of pharmacy and pharmaceuticals. The eRecept system as one of the components of e-health has proven its value, especially during the COVID-19 pandemic. The FG participants supported the development of other e-health tools.

*“E-recipes are a huge simplification for us. But I would imagine there’s even more to it. For example requests form for medical devices. “(FG II-I7).*

Stakeholders disagreed on regulations in the area of delivery-service dispensing of prescription drugs and related services of pharmacists.

*“… with telemedicine or any digitization, the patient should be able to go through the whole process from start to finish, from a consultation, from a diagnosis to e-prescription, to eventually having the medicine delivered to their home.” (FG I-I1).*

*“I cannot imagine that we will start turning our pharmacists into couriers. This is not the route we want to go down.” (FG II-I4).*

On the contrary, all agreed on the need to innovate the relationship between pharmacists, doctors and patients in the context of the introduction of telemedicine solutions. In particular, they supported the expansion of pharmacists’ competences to include consultation services and closer collaboration with physicians.

*“The pharmacist, together with the doctor as partner, caring about the patient’s health, in order to solve the problem. It’s about the doctor and pharmacist working together to benefit the patient’s health.” (FG II-I3).*

#### 3.2.7. Reimbursement of telemedicine solutions

Healthcare digitalization tools can significantly contribute to the necessary higher cost-effectiveness of healthcare, and thus respond to long-term and current challenges not only in the Czech healthcare system. FG participants agreed that telemedicine interventions in terms of support for reimbursement from public health insurance must be defined with regard to their effectiveness, costs, and added value for providers and patients.

*“Avoid a blanket introduction of telemedicine. Introduce specialized telemedicine where it counts, where it makes sense both from the patient’s point of view and from an economic point of view.” (FG I-I3).*

The introduction of telemedicine procedures into the public health insurance system should be allowed on the basis of standard procedure and opposition.

*“Insurance companies, in cooperation with experts, but also with those who offer those particular types or particular ways of telemedicine solutions, should determine in what form they will enter into reimbursement.” (FG III-I2).*

In relation to reimbursement from public health insurance or direct patient payment, a distinction should be made between telemedicine procedures that bring an improvement in patient comfort and those that bring a therapeutic benefit.

*“Telemedicine is not only wanted by technology providers and producers. Patients want it too. And it has to be said that some of the care that patients choose to receive in this way will not be covered by public health insurance. For example, if they want a consultation in the evening.” (FG III-I1).*

According to stakeholders, digital communication between health insurance companies and health service providers should also be supported as an important tool for an effective control system.

*“To help find any inefficiencies in the system, reporting of healthcare or its provision from public health insurance, and it will also facilitate the auditing process to ensure that what should be covered is really covered.” (FG III-3).*

#### 3.2.8. Education and competences of health professionals

New models of remote healthcare require healthcare professionals to acquire the necessary ICT skills. FG participants agreed on the need to integrate telehealth and digital skills into the educational programs of health professionals in undergraduate and postgraduate education.

*“We have a huge deficit in communication skills training in medical faculties. This cuts across all disciplines and it is terribly important that communication skills are developed with digital in mind.” (FG I-I1).*

Stakeholders agreed that the use of ICT in health care provision also allows for a more active involvement of non-medical health professionals and recommended their greater involvement in the implementation of telemedicine.

*“The issue of incorporating video consultations or those ways of providing healthcare into the work of healthcare providers. It does not always have to be physicians. Somewhere, general nurses or other types of health professions will suffice.” (FG III-I6).*

A more active involvement of non-medical health professionals in telecare in the future will not be possible without defining their new competences. However, this will require changes in the law and in training programs for individual disciplines.

*“One of the key issues is what a nurse can do and what a doctor must do. It will be very discipline-specific.” (FG III-I6).*

*“The unpreparedness of the Czech Republic is also in the competencies. So that some of the tasks within telemedicine can be done by a non-physician. But they cannot even do that because we have not prepared, for example, nurses to have the competence to do some things.” (FG I-I2).*

#### 3.2.9. Patient education and awareness

The use of remote healthcare using ICT requires a certain level of health and digital literacy from its users.

*“Better health literacy is as much in the physical contact as it is in the delivery of a health service using digital technology. The patient needs a little more information and some better awareness of their rights to be able to possibly refuse the imposed use of digital technology in health service provision. In this sense, some patient education would be helpful.” (FG III-I1).*

FG participants agreed that patients should be educated not only on how ICT can be used in healthcare delivery, including with regard to their safety, but also on what their rights and responsibilities entail.

*“It’s one thing that we need to have some technical standards set, but it’s another thing that the patient, who is the recipient of that service, should be educated on how to use it, and that it all has some limits.” (FG III-I1).*

Stakeholders also pointed to the important role of the state and the role of patient organizations in supporting patient education and increasing patients’ digital skills in using ICT.

*“Patient organizations, in particular, can disseminate information to their members through IT technologies and essentially make that information more available to patients and can convey it in a much more immediate and better way than patients having to look it up on the internet.” (FG III-I8).*

*“I think it’s not just down to patient organizations and patients in general. It is also the role of the state, or perhaps the National Institute of Health, to make sure that awareness – and obligations – of patients’ rights is as widespread as possible.” (FG I-I8).*

#### 3.2.10. Prevention and health promotion

The use of digital technologies, including health apps (mHealth), has a high potential for use in prevention and health promotion.

*“The deployment of these technologies is precisely in the field of primary prevention as well as other prevention programs. In the future, I see the integration of these technologies with smart solutions, for example in the form of smart watches, which gives us hope that we will be able to rehabilitate some patients properly, for example after cancer treatment, to get them back to a better condition.” (FG I-I2).*

FG participants agreed that telehealth solutions increase patients’ compliance and adherence to treatment and strengthen their role in healthcare provision.

*“The benefit of telemedicine is also in increasing compliance, if a patient has the information that the doctor has, then I suppose their curiosity will somehow get them more involved in the game for their own health.” (FG I-I3).*

They also agreed that the use of health apps can also increase motivation for a healthier lifestyle, and serve as a general educational tool to increase health literacy.

*“Telemedicine can be used to both educate the patient regarding their diagnosis and monitor their chronic or acute conditions. Further, there is definitely wide educational potential in personalizing the system through the patient’s mobile app, and I mean very general education, like self-management, lifestyle.” (FG I-I2).*

Explaining the meaning and importance of preventive examinations and supporting projects to use ICT in prevention is perceived by stakeholders as the role of the state. The systemic setting of ICT in prevention should also be supported by health insurance companies.

*“I think that, in general, the need to take more care of ourselves should resonate more in society. This education should also come from insurance companies and from the Ministry of Health.” (FG I-I11).*

### 3.3. Patient panel and comparison

#### 3.3.1. Patient panel

Representatives of patient organizations generally support the development of rules and regulations for the development of telemedicine solutions at all levels of healthcare (24). However, they point out that this model of care brings new roles, relationships and responsibilities, and raises a number of uncertainties and associated expectations and concerns. Patients ask questions, the answers to which will have a major impact on their decision whether or not to trust telemedicine solutions. Representatives of patient organizations identified the following topics as key to the successful development of telemedicine: safe care, protection of confidentiality and privacy, communication in the new environment, uniform conditions for the organization of care, systemic support for telemedicine solutions and digital training for healthcare professionals and patients.

##### 3.3.1.1. Safe care

The lack of a legislative anchor for telemedicine raises patients’ concerns about the legality of care provided by remote access. The lack of development of guidelines for telemedicine increases their legal uncertainty about whether care is being delivered in a professionally correct way (lege artis). It is important for patients to be of sound health. It is also important for patients that the physician’s responsibility for using a telemedicine solution is clearly established. Patients would like to have access to guidelines (information) so that they can learn about in which situations the use of telemedicine is appropriate and safe.

*“What can I even address remotely? Is telemedicine safe? Will a telemedicine exam be as good as an in-office exam? Might the doctor miss something? Who is responsible for the care I choose?” (Questions from the patient panel).*

##### 3.3.1.2. Confidentiality and privacy

Patients are concerned about the safety of the technology used, the security of the data transmission, and the quality of care provided if conditions (standard) are not set for the technical equipment. Clearly defined rules for the provision of telemedicine services are important to patients with regard to privacy and online access to their health data. Patients would welcome a database of secure technological solutions, including health applications.

*“How is the transmission of my data secured? Where does the data from my measuring device go? What happens to my data? Who is my data shared with? Can it be misused? How will my privacy be secured on the provider side? Who else may participate in the telemedicine service?” (Questions from the patient panel).*

##### 3.3.1.3. Communication in a new environment

Telemedicine increases demands on communication between doctors and patients. Patients are concerned that a lack of communication may lead to a lower quality of care. It is important for patients that there is a single standard for digital communication that they would like, with their doctors, to participate in creating. They consider it crucial that the rules for the provision of telemedicine services respect the autonomy of patient decision-making. They would welcome guidance and education on how to communicate with physicians in the online environment. They also support the practice of completing structured guidance questionnaires prior to an appointment, to enable them to better prepare for their appointment with the doctor.

*“What should I say to the doctor and how? What should the doctor ask me? How will the telemedicine exam be different? How will the doctor identify me? How will the patient’s informed consent be secured? How will my right to make decisions about my care be assured?” (Questions from the patient panel).*

##### 3.3.1.4. Uniformly-set conditions for the organization of care

Different approaches and conditions between each of the providers in setting up and using telemedicine solutions make the system unclear for patients. Patients are concerned about providers mandating certain ICT configurations they will have to manage. It is important for patients that providers make information public on the scope of the telemedicine services to be provided. They would welcome user guides on technology solutions and plainly support online booking systems.

*“Do I need to use telecare? I do not have the technical equipment that telemedicine requires, so will the service be unavailable to me? Will telemedicine work the same everywhere? Why will not my doctor answer the phone? Why do not booking systems work in health services? How can I find out which doctor provides telemedicine services?” (Questions from the patient panel).*

##### 3.3.1.5. System support for telemedicine solutions

The absence of conditions for the entry of telemedicine solutions into reimbursement raises concerns on the part of patients about whether providers will be motivated to use them. Patient organizations clearly support the inclusion of telemedicine in health insurance reimbursement, and want to be part of a transparent process to set up a reimbursement system for telemedicine procedures and telemedicine solutions. Patients also want to decide how medicines are dispensed, and support systemic solutions to enable remote dispensing.

*“Which telemedicine procedures are in the reimbursement system? How will the entry of telemedicine procedures into the reimbursement system be evaluated? Will patients (patient organizations) be able to influence which telemedicine procedure should be included in the system? If I have an e-prescription, why do not I have ‘e-medicine’? Why cannot I get my prescription medication delivered to my home?” (Questions from patient panel).*

##### 3.3.1.6. Digital training for healthcare professionals and patients

The low level of digital literacy of patients and healthcare professionals raises concerns about the safe use of ICT, and the poor accessibility, and the quality of remote healthcare. Patients support educational and motivational programs to acquire and expand their ICT skills. They stress that the patient’s perspective should not be neglected in the education of health professionals. Thus, patient organizations want to be involved in programs to increase digital literacy of citizens, patients, and healthcare professionals.

*“How can I learn to work with new technologies? What new skills will doctors and patients need to learn?” (Questions from patient panel).*

#### 3.3.2. Comparison of patient’s and other stakeholders’ perspective

A comparison of the FG outputs and the patient panel’s opinions showed that there is consensus between the stakeholder and patient conclusions in most of the key areas described by the research team. Both groups agree on the identification of key issues and challenges for the implementation of telemedicine in the Czech Republic.

In the area of communication and data sharing, patients came up with concrete solutions to eliminate their concerns about miscommunication or lack of communication in a new and unfamiliar environment. They propose the creation of rules (standards) for digital communication between patients and doctors, and also rules (standards) for shared decision making (informed consent) in the online environment.

It also showed that in each key area, another patients’ perspective can be identified, which appropriately complements or even extends the stakeholders’ conclusions on the process of telemedicine implementation. This perspective was identified (described) by the research team as “patient interest” (see Table 3).

### 3.4. Recommendation

The research team formulated recommendations that would strengthen patient and public confidence in telemedicine interventions, taking into account the possibilities of collaboration between patient organizations and healthcare professionals in the development of communication (online) strategies and their involvement in the processes of telemedicine implementation. Within the process of telemedicine implementation, the proposed recommendations can be used by individual stakeholders separately or interconnected at different levels of healthcare management.

- Involve patients in the development of telemedicine rules, decision-making, and evaluation processes for reimbursement of telemedicine solutions and certification of healthcare applications (support the development of patient involvement strategies).
- Ensure that patients have systematic (open) access to information on telemedicine interventions and their suitability and safe use for individual therapeutic areas, and safe telemedicine solutions including health apps.
- Provide patients with information on telemedicine interventions at the individual provider level, including the definition of a time pool for telemedicine by specific providers.
- Make user guides for telemedicine solutions available to patients by individual providers, including the provision of tech support.
- Promote collaboration between healthcare professionals and patient organizations to develop rules for safe and effective communication in the online environment (digital communication standards).
- Development, in collaboration with patient organizations, of targeted educational programs for patients and the public to increase the level of digital health literacy and a better understanding of the telemedicine care provided.
- Include patient interest in targeted interventions to educate health professionals on telehealth.
- Take into account technical inequalities and ensure wide accessibility of telemedicine services, while preserving patients’ freedom of choice. Promote the ethical adoption of digital health technologies in the provision of remote healthcare.

## 4. Discussion

The successful implementation of any technology into the healthcare system depends largely on the trust of its end users, i.e., the public and patients (28). Telemedicine is a new service in healthcare, and therefore understanding the attitudes that patients have towards it is important to facilitate its adoption (29).

Similar to other authors (11, 23), we base our research on the premise that involving patients in implementation processes at all levels of the health system as key users of health services contributes to protecting their interests, improving the quality and safety of services, and making them patient-centered.

The greater experience with telemedicine in the Czech Republic, reinforced by the COVID-19 pandemic, has contributed to the fact that a wider introduction of telemedicine elements in different healthcare fields is already generally supported by patients (12, 30). Our research also confirmed the high level of patient acceptance of telemedicine interventions, similar to the level of acceptance in foreign studies (31, 32).

Although use of telemedicine has declined from its peak during the pandemic, it remains well above pre-pandemic levels (33, 34). However, it appears that traditional health care regulation is not sufficient to address the (legal, social, and ethical) issues associated with innovative technologies (29). Thus, individual health systems are seeking a balance between in-person and virtual care delivery (32). Patients are also adapting to the new model of “hybrid care” (mixed care), and it is important to understand their concerns and feelings in order for telemedicine to continue to develop (35).

The results of our research have shown the unpreparedness of the Czech healthcare system to deliver ICT-enabled telemedicine services across the full spectrum of stakeholders [similarly (36)]. In agreement with patients, stakeholders identified regulatory uncertainty as the main barrier to telemedicine integration, leading to the incoordination of telemedicine implementation in the Czech Republic. Thus, the absence of legislative regulations and other (disciplinary and organizational) guidelines for telemedicine can generate a number of problems in the patient–doctor relationship (37, 38), including ethical ones (27, 39). This may also lead to inequalities in access to healthcare (3, 40) and affect the overall quality of care provided (41).

Involving patient organizations in our research allowed us to understand their values, beliefs, knowledge, experiences, motivations and attitudes in relation to telemedicine. Thusly, “patient interest” in all key areas of telemedicine implementation could be identified. With regard to patient interest, it became clear that patients want: 1. a predictable and reliable framework that provides them with certainty and security in the provision of telemedicine services, 2. telemedicine solutions that increase the availability and efficiency of the care provided and also bring convenience (e.g., in terms of time savings), and 3. user-friendly and simple solutions. At the same time, they want to understand the new environment.

It has been shown that patients want to be active participants in the process of digital innovation, including its practical implementation (e.g., collaborating with physicians to create rules for shared decision-making) (42). Telemedicine provides an ideal environment for shared decision-making, which is essential for building patient-centered care (43). Involving patients in collaboration with physicians can lead not only to improved communication in the delivery of online care, but also to improved quality of life, as well as empowerment of patients (44).

Recommendations developed by the research team that reflect the patient’s interest can be implemented at three levels – at the health system level (policy), at the institutional level (providers, insurers), and at the community level (patient organizations, regions). The implementation of these recommendations at each level can intersect and influence each other.

In this context, Otto et al. (45) point out that communities play a key role in the successful scale-up of telemedicine interventions. The community can actively influence and encourage individuals to adopt telemedicine, for example, by conducting awareness campaigns or creating support programs for disadvantaged community members. However, the community itself is also affected by various factors (e.g., legal and regulatory constraints) that influence its readiness for telemedicine. In countries with a developed and institutionalized patient movement, including the Czech Republic, it is patient organizations that can represent the community level. Other authors, for example Zhang et al. (46), point out that telemedicine stakeholders should strengthen intersectoral collaboration to incorporate population preferences and entrench the service in the healthcare system.

The readiness of patient organizations for telemedicine initiatives, as one of the key communities, can help bridge the gap between individual patient decisions (attitudes) to adopt telemedicine and system-wide efforts to implement them (45).

Based on the results of our research, future studies could look more closely at the barriers and motivators to patient organization involvement in telemedicine adoption. Consideration of ‘patient interest’ in other phases of telemedicine implementation could also be explored, including with respect to individual telemedicine interventions at different levels of the health system.

## 5. Limits of the research

Our research involved a wide range of stakeholders, including patients. This gave us a comprehensive view of the implementation of telemedicine in the Czech Republic. A limitation of this study is the smaller number of patient panelists. We tried to eliminate this limitation by selecting patient representatives from an umbrella organization who have been active in the patient movement for a long time and also have experience with telemedicine at the individual and system level. Another limitation may be the subjective aspect in identifying “patient interest,” which we tried to avoid by having it identified by a pair of team members. We avoided the subjectivity in making recommendations by involving the whole team in their formulation based on a panel discussion of experts.

## 6. Conclusion

In our research, the basic pillars of telemedicine implementation in the Czech Republic were defined. Specific activities within each pillar should be interrelated. Therefore, the development of a state-coordinated strategy and implementation plan for telemedicine is crucial for the further development of telemedicine. All stakeholders, including patients, should be involved in the development and implementation of this strategy for the development of telemedicine, allowing their needs, priorities and expectations to be taken into account. Involving patient organizations can be an effective way to involve patients in initiatives related to the development and implementation of telemedicine. Patient organizations can thus become the link between telemedicine policy making and implementation at the individual level of healthcare provision.

## Data availability statement

The raw data supporting the conclusions of this article will be made available by the authors, without undue reservation.

## Ethics statement

Ethical review and approval was not required for the study of human participants in accordance with the local legislation and institutional requirements. All research participants provided informed consent to participate in this study.

## Author contributions

JT and KD led the conception and design of this paper, collected and analyzed data, and solicited financial contributions. JT, KD, ZD, and AT formulated recommendations. All authors contributed to the article and approved the submitted version.
